# Genome adaptation to chemical stress: clues from comparative transcriptomics in *Saccharomyces cerevisiae *and *Candida glabrata*

**DOI:** 10.1186/gb-2008-9-11-r164

**Published:** 2008-11-24

**Authors:** Gaëlle Lelandais, Véronique Tanty, Colette Geneix, Catherine Etchebest, Claude Jacq, Frédéric Devaux

**Affiliations:** 1 Equipe de Bioinformatique Génomique et Moléculaire, INSERM UMR S726, Université Paris 7, INTS, 6 rue Alexandre Cabanel, 75015 Paris, France; 2 Laboratoire de Génétique Moléculaire, CNRS UMR 8541, Ecole Normale Supérieure, 46 rue d'Ulm, 75230 Paris cedex 05, France; 3 Plate-forme transcriptome IFR 36, Ecole Normale Supérieure, 46 rue d'Ulm, 75230 Paris cedex 05, France; 4 Current address: MTI, Bât. Lamarck, 35 rue Hélène Brion, 75205 Paris Cedex 13, France

## Abstract

**Background:**

Recent technical and methodological advances have placed microbial models at the forefront of evolutionary and environmental genomics. To better understand the logic of genetic network evolution, we combined comparative transcriptomics, a differential clustering algorithm and promoter analyses in a study of the evolution of transcriptional networks responding to an antifungal agent in two yeast species: the free-living model organism *Saccharomyces cerevisiae *and the human pathogen *Candida glabrata*.

**Results:**

We found that although the gene expression patterns characterizing the response to drugs were remarkably conserved between the two species, part of the underlying regulatory networks differed. In particular, the roles of the oxidative stress response transcription factors ScYap1p (in *S. cerevisiae*) and Cgap1p (in *C. glabrata*) had diverged. The sets of genes whose benomyl response depends on these factors are significantly different. Also, the DNA motifs targeted by ScYap1p and Cgap1p are differently represented in the promoters of these genes, suggesting that the DNA binding properties of the two proteins are slightly different. Experimental assays of ScYap1p and Cgap1p activities *in vivo *were in accordance with this last observation.

**Conclusions:**

Based on these results and recently published data, we suggest that the robustness of environmental stress responses among related species contrasts with the rapid evolution of regulatory sequences, and depends on both the coevolution of transcription factor binding properties and the versatility of regulatory associations within transcriptional networks.

## Abstract 2

Comparative transcriptomics of *Saccharomyces cerevisiae* and *Candida glabrata* revealed a remarkable conservation of response to drug-induced stress, despite underlying differences in the regulatory networks.

## Background

As evolutionary changes frequently involve modifications to transcriptional regulatory programs, the integration of gene expression data into classic cross-species comparisons based on protein or DNA sequence similarity is a powerful approach likely to improve our understanding of phenotypic diversity among organisms. Sequence similarity between genes or proteins is not always proportional to the conservation of function during evolution [1,2] and investigations of the conservation of gene expression patterns are, therefore, useful for precise determinations of function [3-5]. Comparative functional analyses have been made possible by the accumulation of large-scale gene expression datasets for a large number of organisms, due directly to the exponential increase in the number of species for which whole genome sequences are available [6,7]. The development of methodologies for comparing genome-wide gene expression data between species has been challenging, and several computational approaches have been proposed in the past five years for the integration of cross-species expression and sequence comparisons [2,8-12]. Combining sequence and expression data appeared to be useful for improving functional annotation of genes [13,14], for refining modules of homologous genes in different organisms [15,16] or for increasing our understanding of the regulatory relationships between genes among species [17,18].

Pioneering studies focused on evolutionarily distant model organisms, for which all the publicly available microarray data were combined into a single dataset [8,9]. These studies gave interesting results, demonstrating the potential of cross-species comparisons based on expression data. However, the evolutionary distance between the compared species and the combination of unrelated expression data limited the conclusions to the characterization of transcriptional modules consisting of large numbers of genes with very high levels of sequence conservation and very highly correlated expression patterns. To increase the accuracy of investigations of the evolution of genetic networks, we would like, in an ideal case, to: compare selected microarray experiments that are as similar as possible for all species considered; and compare species separated by an optimal evolutionary distance, that is, species sharing a high level of orthology but with different lifestyles and physiological properties [11]. In this respect, the hemiascomycete phylum constitutes a valuable model. Yeast species have evolved in niches with constantly varying nutrient availability and growth conditions, and have thus had to develop sophisticated mechanisms for controlling genome expression. More than ten yeast species have now been fully sequenced [19,20], opening up new possibilities for studying the adaptation of transcriptional networks to environmental constraints over a progressive evolutionary scale spanning 400 million years [11,21].

We present here a comparative analysis of the transcriptional programs driving the chemical stress response in two evolutionarily close yeast species, *Saccharomyces cerevisiae *and *Candida glabrata *[20]. *C. glabrata *is a pathogenic yeast and the frequency of systemic infections with this yeast is increasing, perhaps due to the extensive use of azole antifungal agents, to which *C. glabrata *may be resistant [22,23]. In contrast to *S. cerevisiae*, in which genome expression has been extensively studied, very few functional genomic studies have yet been carried out for *C. glabrata*, and very little is known about its drug resistance pathways [24,25]. Most functional annotations of *C. glabrata *genes are currently based on sequence similarity with genes of *S. cerevisiae *that have been well characterized functionally. One clear challenge for comparative functional genomics concerns the extension of our considerable knowledge of *S. cerevisiae *genetic networks to other yeasts, such as *C. glabrata*. With this goal in mind, we focused on the early genomic events characterizing the stress response induced by benomyl, an antifungal agent that inhibits cell growth during mitosis.

In *S. cerevisiae*, benomyl has been shown to activate an oxidative stress response primarily dependent on the transcription factor ScYap1p [26]. Our global analyses showed that this drug induces the expression of orthologous gene pairs involved in oxidative stress responses similarly in both species, suggesting a high degree of conservation of the corresponding pathways in these two species. Combining the differential clustering algorithm (DCA) [10] with promoter sequence analyses, we observed that, despite the highly conserved patterns of expression of genes regulated by benomyl in the two species, the transcriptional pathway related to the transcription factor Yap1p appeared to have substantially changed. Experimental assessment of the genes actually controlled by Cgap1p, the functional homolog of ScYap1p in *C. glabrata*, indicated that even if Cgap1p retained an important role in the benomyl response, this function was less important than that of ScYap1p in the *S. cerevisiae *benomyl response. Interestingly, the Yap1 response element (YRE), which is the most enriched in the promoters of Cgap1p target genes, is only marginally present in the promoters of Yap1p-dependent genes. Finally, our data are consistent with a divergence of the Cgap1p recognition sites from the preferred binding sequences for ScYap1p. In terms of the oxidative stress response, this divergence of the promoter regions between *S. cerevisiae *and *C. galabrata *is counterbalanced by coevolution of the DNA binding sites of transcription factors and by the flexibility of transcriptional networks, ensuring the robustness of the genomic response of cells to hostile chemical environments.

## Results

### Transcript profiling with identical experimental conditions in both yeast species

#### Benomyl dose and measurement times

We carried out microarray analyses of the transcriptome responses of *S. cerevisiae *and *C. glabrata *following identical treatments with the antifungal agent benomyl [27]. Both yeast strains were subjected, in parallel, to the growth conditions defined in our previous study [26]: 20 μg/ml benomyl for 2, 4, 10, 20, 40 and 80 minutes. Labeled cDNA from treated cells was hybridized with *S. cerevisiae *or *C. glabrata *microarrays in the presence of cDNA from mock-treated cells as a competitor.

#### Global analysis of changes in gene expression shows quantitatively similar transcriptional responses

**Figure 1:**
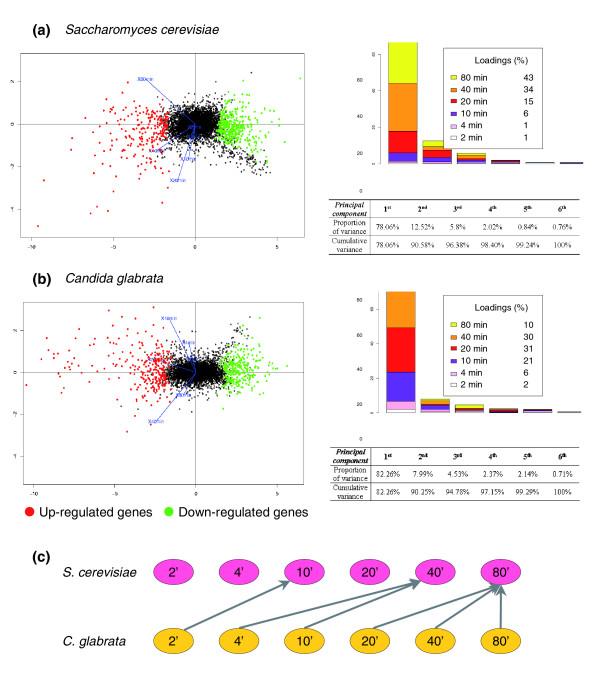
**PCA analysis of the time-course responses of *S. cerevisiae *and *C. glabrata *transcriptomes to chemical stress**. Microarray results were analyzed by PCA. The **(a) ***S. cerevisiae *and **(b) ***C. glabrata *datasets were examined independently. The panels on the left show biplots of the PCA results. Points represent genes. The horizontal axes correspond to the first principal component (PC_1_), accounting for 78% of the total variance in *S. cerevisiae *and 82% in *C. glabrata*. Vertical axes correspond to the second principal component (PC_2_), accounting for 13% of the total variance in *S. cerevisiae *and 8% in *C. glabrata*. Initial time vectors are shown in blue and genes significantly up- and down-regulated are shown in red and green, respectively. The panels on the right show the variability accounted for by each component. Each panel also shows the loadings of initial time vectors on the first principal component (PC_1_). In both species, the first two principal components account for more than 90% of the global variance in the microarray datasets. **(c) **Graphical representation of the relationships between the time points in the two species studied here. In each species, the time point expression measurements are represented by nodes and arrows connect experiments with the highest correlation values (Additional data file 1) for cross-species correlation values between different time points).

We used principal component analysis (PCA) to obtain a global view of the changes in gene expression occurring in response to the addition of benomyl. This multivariate statistical technique allowed us to identify new variables - the principal components (PCs) - that are linear combinations of the original time vectors and account for the largest proportion of the variance of the data. A complete description of PCA can be found in [28]. The results of independent PCAs for *S. cerevisiae *and *C. glabrata *benomyl expression data are presented in Figure 1a, b. In both yeasts, more than 90% of the observed variability was accounted for by the first two principal components (Figure 1a, b, right panels). These were used for the simultaneous representation of all the microarray results (Figure 1a, b, left panels). The resulting PCA diagrams were very similar, suggesting that benomyl had a similar impact on the transcriptomes of *S. cerevisiae *and *C. glabrata*. Interestingly, the dominant component PC_1 _consisted primarily of time vectors 80 and 40 minutes in *S. cerevisiae *(loadings were 43% and 34%, respectively), whereas in *C. glabrata*, PC_1 _consisted primarily of the earlier time vectors 40 and 20 minutes (loadings were 30% and 31%, respectively). Such a result meant that the maximal expression variability in *S. cerevisiae *was reached at later times compared with that of *C. glabrata*, and was in agreement with pair-wise correlation values calculated between different time points in different species (Figure 1c; Additional data file 1). In summary, our PCA and cross-species correlation analyses stated that the two benomyl responses were quantitatively similar, although the *C. glabrata *response was faster than that of *S. cerevisiae*.

#### Definition of lists of genes displaying significant changes in expression in response to benomyl

From all the genes for which expression data were available, we identified genes whose expression was significantly modified after benomyl addition, using the significance analysis of microarrays (SAM) procedure [29]. In total, 228 genes in *S. cerevisiae *and 272 genes in *C. glabrata *were found to be up-regulated, whereas 379 genes in *S. cerevisiae *and 298 genes in *C. glabrata *were found to be down-regulated (Additional data file 2).

#### Construction of an orthology table for expression comparisons

To address the evolution of transcriptional programs involved in chemical stress responses, it was important to determine whether 'orthologous' genes in the two yeasts were similarly involved in the biological processes comprising the benomyl stress response. We inferred orthology relationships between the complete genomes of *S. cerevisiae *and *C. glabrata*, using the INPARANOID algorithm [30]. We found orthology links in *S. cerevisiae *for almost 90% of the *C. glabrata *genes. Such a result pointed out the high coding sequence similarity between the two genomes [21]. Orthologous gene pairs for which at least one gene (in one species) displayed a change in expression in response to benomyl stress were then identified. In total, 718 orthologous gene pairs were selected and used as the kernel for cross-species comparisons.

### Global comparison of transcriptional networks, based on DCA and promoter analyses

#### DCA reveals significant conservation of coexpression relationships between orthologous genes

**Figure 2:**
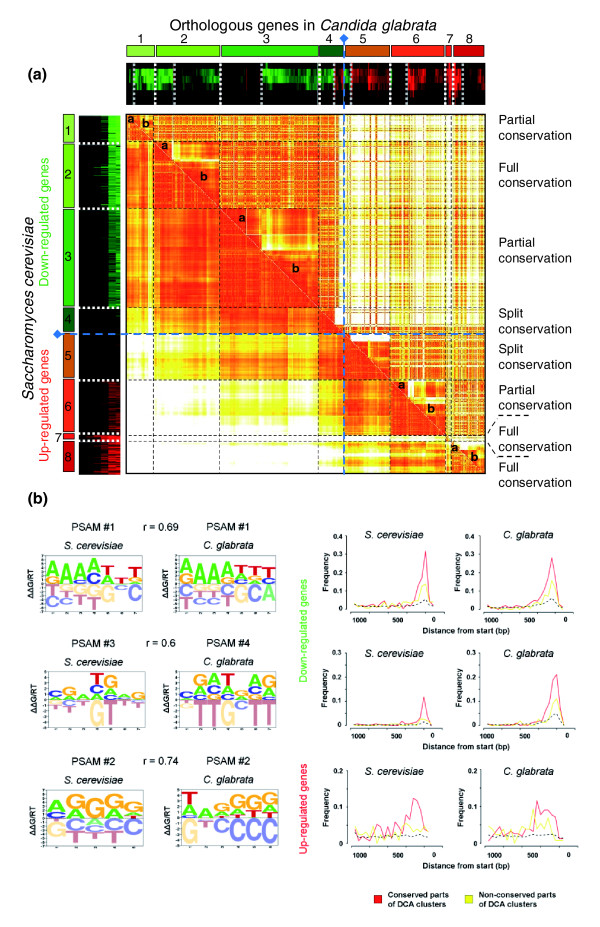
**Global comparison of the *S. cerevisiae *and *C. glabrata *chemical stress responses based on DCA and MatrixREDUCE analyses**. **(a) **We analyzed 718 orthologous gene pairs for which at least one gene displayed a change in expression in response to benomyl stress using the DCA method [10]. The DCA cluster pairs of orthologous genes according to their expression in each species (see Additional data file 3 for a complete description of the DCA method). *S. cerevisiae *was used as the 'reference' yeast whereas *C. glabrata *was used as the 'target' yeast. Eight clusters were obtained after primary hierarchical clustering using the *S. cerevisiae *expression profiles. Each cluster was then split into two subclusters (labeled 'a' and 'b') after secondary hierarchical clustering using the *C. glabrata *expression profiles. Gene expression profiles are indicated with a color code [80]: green for down-regulated genes and red for up-regulated genes. Based on the mean correlations between gene expression levels within and between 'a' and 'b' subgroups, eight conservation clusters were defined: three clusters displaying 'full conservation' (clusters 2, 7 and 8); three clusters displaying 'partial conservation' (clusters 1, 3 and 6); and two clusters displaying 'split conservation' (clusters 4 and 5). The biological relevance of these clusters is discussed in Additional data file 4. **(b) **Three pairs of PSAMs identified with the MatrixREDUCE algorithm [31] and that exhibited significant Pearson correlations (r > 0.6) are shown in the panel on the left. They correspond to specific regulatory sequences that are evolutionarily conserved between *S. cerevisiae *and *C. glabrata*. The panel on the right shows the frequency of occurrence of PSAM in 50 bp windows of the gene clusters identified with the DCA. Background genomic frequency is indicated in black (dashed line); the frequency in conserved parts of DCA clusters is indicated in red (clusters 1b, 2 and 3b for down-regulated genes, and clusters 6b, 7 and 8 for up-regulated clusters); and the frequency in non-conserved parts of DCA clusters is indicated in yellow (clusters 1a, 3a and 4 for down-regulated genes, and clusters 5 and 6a for up-regulated clusters). Together, the DCA and MatrixREDUCE results allowed the identification of a set of orthologous genes whose expression and regulation is conserved between the two species examined here.

DCA [10] was used to investigate the evolutionary properties of clusters of genes coexpressed in one or both of the yeast species. This approach systematically characterizes the conservation of coexpression patterns between genes, by means of an original method involving the clustering of orthologous gene pairs according to their behavior in each species (see Materials and methods; Additional data file 3). Briefly, DCA is a two-step procedure involving: the definition of transcriptional modules of coexpressed genes in one species (referred to as the 'reference' species); and the definition of two subgroups of genes (named '*a*' and '*b*') in each module, using the expression data for the orthologous genes in the second species (referred to as the 'target' species). Finally, the similarity of expression profiles in subgroups *a *and *b *is estimated, calculating three correlation values corresponding to the mean correlation of gene expression measurements within and between subgroups *a *and *b*. Depending on these correlation values, the modules will be classified in the 'full', 'partial', 'split' or 'no' conservation categories (Figure 2a). In the particular case of benomyl response, eight coexpression clusters were defined on the basis of the gene expression data for *S. cerevisiae*. Based on expression measurements for orthologous genes in *C. glabrata*, three of these modules were annotated as displaying full conservation (cluster 2 = 132 genes, cluster 7 = 12 genes and cluster 8 = 66 genes), three modules were annotated as displaying partial conservation (cluster 1 = 58 genes, cluster 3 = 197 genes and cluster 6 = 110 genes) and two modules were annotated as displaying split conservation (cluster 4 = 51 genes and cluster 5 = 92 genes). The different transcriptional modules and their biological properties are described in Additional data file 4 and complete gene lists in each module can be found in Additional data file 5. Taken as a whole, the full conservation clusters (2, 7 and 8) and the conserved parts of the partial conservation clusters (cluster 1b = 42 genes, cluster 3b = 112 genes and cluster 6b = 75 genes) demonstrated a strong evolutionary conservation of the transcriptional pathways driving the benomyl response in the two species, more than 60% of the orthologous gene pairs conserving their co-expression properties.

#### Promoter analyses identify three conserved transcriptional pathways

We investigated the regulatory processes governing the benomyl stress response by combining our time course expression data with comparative analyses of the promoter sequences. In each species, we applied the MatrixREDUCE algorithm [31] and identified significant position-specific affinity matrices (PSAMs) that represent the sequence-specific binding affinity of potential transcription factors. Complete results obtained with MatrixREDUCE are shown in Additional data file 6. Most notably, we could identify three pairs of PSAMs between *S. cerevisiae *and *C. glabrata *that exhibited significant Pearson correlations (r > 0.6); these are shown in Figure 2b (left panel) and correspond to specific regulatory sequences that are evolutionary conserved. The AAAATTT (PSAM 1 in *S. cerevisiae *and PSAM 1 in *C. glabrata*) and CGATGAG (PSAM 3 in *S. cerevisiae *and PSAM 4 in *C. glabrata*) motifs correspond to motifs named rRPE and PAC, respectively [32,33]. They have been identified in the promoters of genes repressed during the environmental stress response, most of which encode ribosomal proteins or proteins involved in ribosome biogenesis and rRNA processing [34]. The AGGGG motif (PSAM 2 in *S. cerevisiae *and PSAM 2 in *C. glabrata*) correspond to the stress response element (STRE) identified in the promoters recognized by the environmental stress response factors Msn2p and Msn4p [35]. This inter-species conservation of DNA motifs involved in both down- and up-regulation of genes responding to benomyl indicate that at least three identical transcriptional pathways were involved in the chemical stress response in *S. cerevisiae *and *C. glabrata*. To expand on this observation, we examined in more detail the appearance of these three motifs in the promoters of the orthologous genes that we analyzed with DCA (Figure 2a), making a distinction between orthologous pairs that belong to the conserved and the non-conserved parts of the DCA clusters (Figure 2b, right panel). For each motif, we could observed that its position relative to that of the open reading frame (ORF) start codon was highly conserved between the two yeasts and that its frequency was systematically higher in the conserved DCA clusters than in the non-conserved parts. In summary, the combination of DCA and MatrixREDUCE efficiently extracted a set of orthologous genes whose expression and regulation is conserved between the two species examined here.

### Comparative analysis of the Yap1p-mediated transcriptional modules controlling the benomyl stress response in *S. cerevisiae *and *C. glabrata*

#### ScYap1p and Cgap1p have different impacts on benomyl response

**Figure 3:**
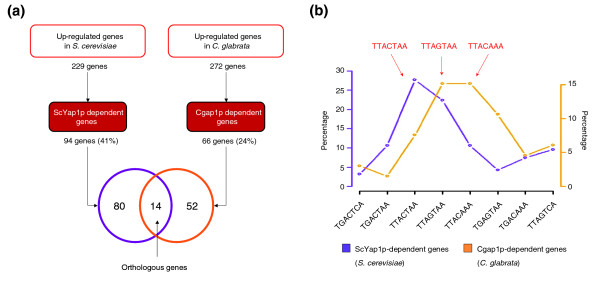
**Comparative analysis of Yap1-mediated transcriptional modules**. **(a) **Genes up-regulated during the time course of benomyl treatment were assigned to two groups as a function of their regulation by the transcription factors ScYap1p in *S. cerevisiae *(ScYap1p-dependent genes) and Cgap1p in *C. glabrata *(Cgap1p-dependent genes). In *S. cerevisiae*, the ScYap1p transcription factor accounts for 41% of the genes induced during benomyl stress, whereas, in *C. glabrata*, the transcription factor Cgap1p accounts for 24% of the genes induced during the benomyl stress response. **(b) **Eight versions of the YRE have been described in previous studies (TGACTCA [39], TGACTAA[38], TTACTAA[38], TTAGTAA [37], TTAGTCA[38], TGACAAA[40], TGAGTAA [40]and TTACAAA [40]). We looked for these motifs in the upstream regions (from nucleotides -600 to -1, direct strand) of up-regulated genes during the benomyl stress response. The percentages of genes with a YRE in their promoter are shown here. In *S. cerevisiae*, the motifs TTACTAA and TTAGTAA appeared to be the more frequent in the promoters of genes regulated by ScYap1p, whereas in *C. glabrata*, the motifs TTAGTAA and TTACAAA appeared to be the more frequent in Cgap1p-dependant genes.

The transcription factor ScYap1p has been extensively studied in *S. cerevisiae *as a major regulator of the oxidative stress response [36]. It is one of the main coordinators of the early transcriptional response to benomyl stress [26]. In agreement with these previous reports, our promoter analysis of the *S. cerevisiae *benomyl response identified a PSAM whose consensus sequence (T(G/T)ACTAA) is compatible with the YRE, that is, the binding site of ScYap1p (*S. cerevisiae *PSAM 4; Additional data file 6). A homolog of ScYap1p was recently identified in *C. glabrata *[37]. This homolog, named Cgap1p, restores drug resistance in a *S. cerevisiae yap1Δ *mutant [37] and regulates the expression of *CgFLR1 *in response to benomyl [37]. In *S. cerevisiae*, the *ScFLR1 *gene encodes a transporter of the major facilitator superfamily (MFS) involved in multidrug resistance and is a well known transcriptional target of ScYap1p [38]. The observation that the orthologous genes *CgFLR1 *(in *C. glabrata*) and *ScFLR1 *(in *S. cerevisiae*) may be similarly regulated by Cgap1p and ScYap1p suggested that the Yap1p-mediated transcriptional modules were at least partly conserved between *S. cerevisiae *and *C. glabrata*. However, none of the PSAMs identified in *C. glabrata *exhibited significant Pearson correlation with the *S. cerevisiae *YRE-PSAM (Additional data file 6). To highlight the role played by Cgap1p in the benomyl response of *C. glabrata*, we carried out a series of transcriptome analyses, directly comparing gene expression in the *C. glabrata *wild-type strain and a *CgAP1Δ *strain 20 minutes after benomyl addition. Differential gene expression analysis showed that *CgAP1 *deletion affected the benomyl-mediated induction of 66 of the 272 up-regulated genes (Figure 3a). Therefore, Cgap1p played a key role in the benomyl response by controlling the expression of almost 25% of the genes induced in our experiments. Nevertheless, this contribution was smaller than in *S. cerevisiae*, in which more than 40% of the genes up-regulated by benomyl in this study are regulated by ScYap1p (Figure 3a). Moreover, we could observe that the sets of genes whose benomyl response depends on Cgap1p or ScYap1p are significantly different since only 14 orthologous genes were identified between them. Complete lists of Cgap1p and ScYap1p target genes are supplied in Additional data file 7.

#### Differences in the benomyl response element between *S. cerevisiae *and *C. glabrata*

**Figure 4:**
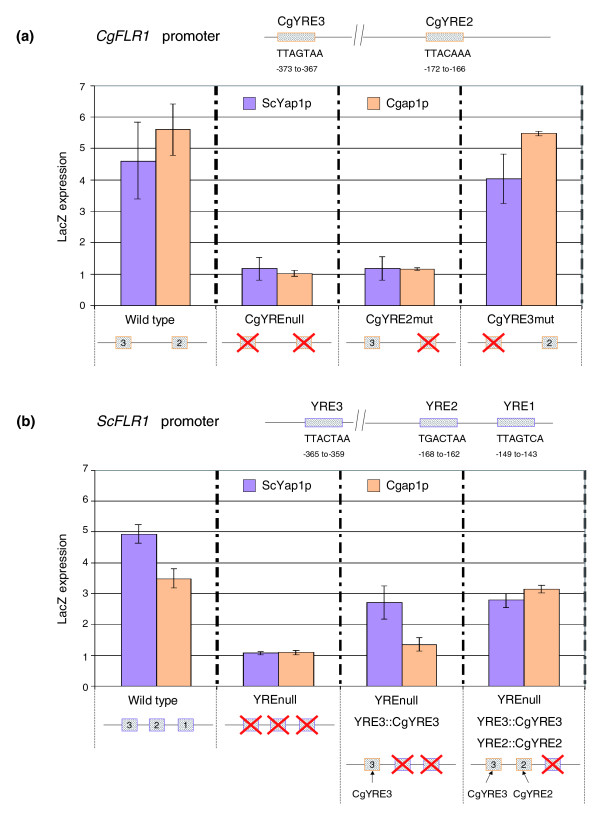
**Functional comparative analyses of ScYap1p and Cgap1p activities *in vivo***. *In vivo *assays of ScYap1p and Cgap1p properties were conducted, using *S. cerevisiae *strains expressing either ScYap1p (purple histograms) or Cgap1p (orange histograms). *LacZ *was used as a reporter gene and was placed under the control of wild-type or mutated versions of **(a) **the *CgFLR1 *or **(b) ***ScFLR1 *promoter regions (see Materials and methods). Descriptions of the mutations performed in YREs are shown in Additional data file 12. *LacZ *expression was measured by real-time quantitative RT-PCR, before and after benomyl treatment (20 μg/ml) for 40 minutes. (a) Only the inactivation of CgYRE2 (TTACAAA) dramatically decreased the benomyl response of *CgFLR1*. In the context of a *C. glabrata *promoter (in this case, *CgFLR1*) TTACAAA acts as the major BRE. (b) The *LacZ *reporter gene was placed under the control of the *ScFLR1 *promoter, in which all three YREs were inactivated and replaced with CgYRE3 and CgYRE2 sequences. To summarize, ScYap1p appeared to be as efficient at the *ScFLR1 *and at the *CgFLR1 *wild-type promoters, whereas Cgap1p was more efficient at the *CgFLR1 *promoter (a, b). Moreover, only the introduction of CgYRE2 was able to restore the full activity of Cgap1p at the *ScFLR1 *mutated promoter, whereas the sole introduction of CgYRE3 sequence restored half of the ScYap1p activity, and the addition of the CgYRE2 sequence did not increase this activity. In the heterologous context of the *ScFLR1 *promoter, CgYRE2 is still the main BRE for Cgap1p, but not for ScYap1p, which prefers CgYRE3.

The observation that a quarter of the *C. glabrata *genes sensitive to benomyl depend on the transcription factor Cgap1p for their upregulation apparently conflicts with the lack of inter-species correlation between YRE-PSAMs. To extend the MatrixREDUCE results, we searched for all published data concerning YRE that had been experimentally characterized. Seven versions of the YRE were found in *S. cerevisiae*: TGACTCA [39], TGACTAA[38], TTACTAA[38], TTAGTCA[38], TGACAAA[40], TGAGTAA [40]and TTACAAA [40]. Little is known about the Cgap1p DNA binding elements in *C. glabrata*. The TTAGTAA motif was recently identified as a potential Cgap1p-binding site, based on its presence in the promoter of the *CgFLR1 *gene [37]. We analyzed the proportion of YREs in the promoter of genes with benomyl stress responses dependent on ScYap1p or Cgap1p (Figure 3b). In *S. cerevisiae*, the ScYap1p-dependent genes mainly contained the TTACTAA motif (28%), and its complementary form, TTAGTAA (22%). This finding is consistent with published reports identifying TTA(C/G)TAA as the major benomyl response element (BRE) for ScYap1p [38]. Different results were obtained for *C. glabrata*. Indeed, the Cgap1p-dependant genes still mainly contained TTAGTAA motifs (15%) but they also contained TTACAAA motifs (15%). By contrast, the TTACTAA motif - the major BRE in *S. cerevisiae *- was present in a relatively low number of the promoters of genes that are regulated by Cgap1p (7%). Finally, a blind search for DNA motifs overrepresented in the promoter sequences of Cgap1p-dependent genes based on the oligomer analysis tool of Regulatory Sequence Analysis Tool (RSAT) [41] also identified TTACAA as the most abundant motif in Cgap1p targets (data not shown). Together, these observations suggest that, in *C. glabrata*, the major BRE is TTACAAA rather than TTA(C/G)TAA. To experimentally verify this hypothesis, we constructed yeast strains expressing either ScYap1p (BY4742) or Cgap1p (BYCgAP1) (see Materials and methods). These strains were transformed with plasmids containing *LacZ *as a reporter gene under the control of wild-type or mutated versions of the *CgFLR1 *promoter (Figure 4a; and Materials and methods). *LacZ *expression was measured by real-time quantitative RT-PCR, before and after benomyl treatment (20 μg/ml, 40 minutes; Figure 4a). We chose *CgFLR1 *as a model target because its induction by benomyl is entirely dependent on Cgap1p in *C. glabrata *[37] and because its promoter contains the two YREs, TTAGTAA (from -373 to -367) and TTACAAA (from -172 to -166), that are the most frequent in the promoters of Cgap1p-dependant genes (Figure 3b). We observed that the inactivation of the TTACAAA motif was sufficient to significantly decrease the benomyl response of *CgFLR1 *in the presence of Cgap1p or ScYap1p (Figure 4a). On the other hand, the inactivation of the motif TTAGTAA had no effect. Such an observation demonstrated that, in the context of a *C. glabrata *promoter (in this case, *CgFLR1*), the TTACAAA acts as the major BRE.

#### Cgap1p and ScYap1p differently 'read' *cis*-regulatory signals in their target promoters

The observation that the major BRE has changed between *S. cerevisiae *and *C. glabrata *opened new questions concerning the binding properties of ScYap1p and Cgap1p. The results presented in Figure 4a suggest that the TTACAAA motif, when placed in the natural context of the *CgFLR1 *promoter, was interpreted as a BRE by both proteins. We then decided to test the effect of this sequence on Cgap1p and ScYap1p activities in the 'heterologous' context of a *S. cerevisiae *promoter. The BY4742 and BYCgAP1 strains were transformed with plasmids containing *LacZ *as a reporter gene under the control of wild-type or mutated versions of the *ScFLR1 *promoter. Briefly, three YREs are present in the *ScFLR1 *promoter, named YRE1-3 (Figure 4b). YRE3 has been shown to be responsible for most of the benomyl response of *ScFLR1*, whereas YRE2 has a minor role and YRE1 no role in this response [38]. As stated above, only two YREs have been described in the *CgFLR1 *promoter. Considering their position from the ATG of the *CgFLR1 *gene, we called them CgYRE3 and CgYRE2 (Figure 4a). The sequence of CgYRE3 (TTAGTAA) is very similar to YRE3 (TTACTAA), whereas CgYRE2 (TTACAAA) is significantly different from both YRE2 (TGACTAA) and YRE1 (TTAGTCA). We first put *LacZ *under the control of a wild-type version of the *ScFLR1 *promoter, in which we then inactivated all three YREs (see Materials and methods). We then introduced the CgYRE3 and CgYRE2 sequences in place of the YRE3 and YRE2 sequences, respectively, and measured the *LacZ *expression. We observed two main differences between the activities of the two transcription factors. First, ScYap1p appeared to be as efficient at the *ScFLR1 *as at the *CgFLR1 *wild-type promoters, whereas Cgap1p was more efficient at the *CgFLR1 *promoter (Figure 4a, b). Second, only the introduction of CgYRE2 was able to restore the full activity of Cgap1p at the *ScFLR1 *mutated promoter, whereas the sole introduction of the CgYRE3 sequence restored half of the ScYap1p activity, and the addition of the CgYRE2 sequence did not increase this activity (Figure 4b). In conclusion, in the heterologous context of the *ScFLR1 *promoter, CgYRE2 is still the main BRE for Cgap1p, but not for ScYap1p, which prefers CgYRE3, that is, the reverse complement of YRE3. This may be due to a sequence or a position effect but, in both cases, it implies that Cgap1p and ScYap1p, although sharing an affinity for the YREs of the *ScFLR1 *and *CgFLR1 *promoters, exhibited clear differences in the way they 'read' the *cis*-regulatory elements present in their target promoters.

## Discussion

### A general protocol for comparing gene expression networks

Comparative analyses of gene expression networks in different organisms are promising for understanding both the molecular basis of phenotypic diversity and the evolution of the interactions between genomes and their environment. One of the main obstacles is the difficulty of comparing data obtained in different experimental conditions between organisms separated by large evolutionary distances. We propose a general protocol for studies of the evolution of genetic networks involved in similar biological processes. We optimized conditions for the integration of expression data into a cross-species comparison by: choosing species from the same phylum and with a high rate of functional orthologous genes; producing experimental data as comparable as possible between species; and sequentially applying a set of complementary bioinformatic approaches to assess the validity of the results (Additional data file 8). We first performed independent analyses of the two sets of microarray data obtained for each species. We carried out PCA to check that the two yeasts displayed comparable transcriptome responses to the benomyl dose used in this study (Figure 1a, b). We then used DCA [10] to compare the transcriptional responses in the two yeast species, based on orthology relationships between genes (Figure 2a). It is important to mention that the method used here to assign orthology links does not really distinguish the 'real' orthologs from the paralog lists. Therefore, what are called, for the sake of simplicity, 'orthologs' in this work, should be understood as 'likely functional orthologs'. DCA was originally applied to large sets of unrelated microarray data, using Gene Ontology as a reference for the definition of groups of genes [10]. We used DCA in a different context; it was applied to a limited set of experimental conditions, with no functional assumptions concerning the relationships between genes. DCA efficiently revealed the structure of the transcriptional modules involved in the stress response. We therefore aimed to decipher the underlying regulatory mechanisms, identifying both transcription factors and the associated regulatory motifs in the promoter sequences of regulated genes. In that respect, the benefit of the MatrixREDUCE algorithm [42] relied on possibilities to identify, from a large pool of potential motifs, those best correlated with the expression data, and motifs common to both yeasts (Figure 2b). Finally, our comparative analysis of Yap1-mediated transcriptional modules (Figures 3 and 4) allowed us to identify interesting properties concerning the evolution of the DNA motifs targeted by ScYap1p (in *S. cerevisiae*) and Cgap1p (in *C. glabrata*), and the DNA binding properties of these two proteins.

### Interplay between the conservation of gene expression patterns and the divergence of regulatory networks

As a case study, we investigated the evolution of the genetic networks controlling the chemical stress responses of the two yeast species *S. cerevisiae *and *C. glabrata*. Unlike previous studies of drug responses in pathogenic *Candida *species [43], this study focused on *C. glabrata *rather than *Candida albicans*, for two reasons: *C. glabrata *is the second leading causal agent of candidiasis in humans; and *C. glabrata *is phylogenetically more closely related to *S. cerevisiae *than it is to *Candida albicans *[20]. The use of *C. glabrata *therefore ensured clear and extensive sequence homology with the model yeast *S. cerevisiae*. Despite a short time delay, our PCA and DCA analyses indicated that transcriptional responses were quantitatively similar in the two yeasts, with the set of genes induced or repressed in both species including more than 400 orthologous gene pairs (60% of the entire set of genes responding to benomyl stress in one or both species). The transcriptional pathways related to the regulatory motifs rRPE, PAC and STRE were found to be conserved, whereas the transcriptional pathway related to the transcription factor Yap1p appeared to have substantially changed. In *S. cerevisiae*, the transcription factor ScYap1p controls the expression of more than 40% of genes up-regulated in the presence of benomyl and a single deletion of the *ScYAP1 *gene is sufficient to abolish this response [26]. In our study, the *C. glabrata *ortholog of ScYap1p, Cgap1p, controlled 'only' 25% of the positive response to benomyl. Reconstructing the evolutionary path of the promoters that 'escaped' the Yap1p regulation in *C. glabrata*, we observed a progressive decrease in the number of these promoters that contained YREs along the *Saccharomyces sensu stricto *evolutionary tree, from 100% in *S. cerevisiae *down to 50% in *S. bayanus *(Additional data file 9). Still, 60% of these promoters have one or more YREs and are actually controlled by the ScYap1p ortholog in the distant yeast species *C. albicans *[44]. These observations suggest that the ancestral regulation of these promoters was dependent on Yap1p. In *C. glabrata*, other combinations of transcription factors may be involved in the oxidative stress response of these genes. The Msn2p/Msn4p transcription factors are good candidates, since a large number of STRE regulatory motifs were observed in the *C. glabrata *genes for which the orthologous genes in *S. cerevisiae *were ScYap1p target genes (data not shown). A different sharing of the work between the seven ScYap1p paralogs, six of which have clear orthologs in *C. glabrata*, could also be investigated.

Together with this quantitative decrease of the regulatory role of Cgap1p, we observed a modification of the Yap1 binding-site sequences present in the promoters of *C. glabrata *genes. Comparative genomics analysis of the YRE in five yeast species (Additional data file 9) showed that the proportions of most of the *S. cerevisiae *YRE motifs are gradually decreasing along the yeast phylogenetic tree, except the TTACAAA and TGACAAA motifs, whose frequencies were significantly higher in *Candida *species (*C. glabrata *and *C. albicans*) than in *S. cerevisiae*. Our functional analyses confirmed that TTACAAA acts as the major BRE in *C. glabrata *promoters (Figure 4). Of note, although the alanine spacer and the second basic cluster of the bZip domain are identical in ScYap1p and Cgap1p, 50% of amino acids in the first basic cluster are substitutions, some of which may account for differences in the DNA recognition properties of the two proteins [37].

The complexity of the evolution of the promoters responding to oxidative stress is nicely exemplified by the *ScFLR1 *gene and its orthologs, *CgFLR1 *in *C. glabrata *and *CaMDR1 *in *C. Albicans*. Indeed, the *FLR1 *response to various sources of oxidative stress, although conserved all along the hemiascomycete tree, relies on different regulatory systems from *S. cerevisiae *and *C. glabrata*, in which the discrimination between H_2_O_2 _and benomyl is based on different *cis*-regulatory elements used by the same transcription factor (this study and [38]), to *C. albicans*, in which the BRE activity has been transferred to a different regulatory pathway (Additional data file 10) [45,46].

## Conclusion

The evolution of transcriptional regulatory networks has made a major contribution to the diversity of life [47-49]. Work in this field was long restricted to analyses of the regulatory networks controlling development in higher eukaryotes, but the recent sequencing of the genomes of more than ten yeast species has placed yeasts at the forefront of evolutionary studies [50]. A phylogeny of functionally important *cis*-regulatory motifs can be established among closely related yeast species [19], but the intimate structure of the promoters and the DNA-binding properties of transcription factors rapidly diverge. A recent study of the genome-wide location of binding sites for the transcription factors Ste12 and Tec1 was carried out in three closely related *Saccharomyces *species and showed that, in this case, the divergence of transcription factor binding sites was associated with a modification in target gene selection, depending on the physiological conditions (pseudohyphal growth versus mating) [51,52]. Progressive divergence of regulatory networks, together with major genome rearrangements, such as entire genome duplication events, led, in hemiascomycetes, to considerable changes in gene expression patterns [53]. However, the divergence of the structure of the regulatory networks is, in many cases, not accompanied by changes in gene expression. For example, the logic underlying mating-type (MAT) target gene regulation is conserved in all hemiascomycetes species examined to date, despite major changes to the regulatory networks controlling MAT gene expression [54]. The control of proteasome expression illustrates another case in which high conservation of gene regulation is connected to a high conservation of the regulatory system, with only a subtle divergence of the corresponding *cis*-regulatory motifs, which co-evolved with the Rpn4p transcription factor DNA binding properties [55]. The case of the oxidative stress response described here turned out to be intermediate. As for the MAT locus, little phenotypic divergence was observed in terms of gene expression patterns or gene co-regulation properties. This high conservation deals with a fast divergence of the promoter sequences, which seems to have been counterbalanced by two phenomena: the co-evolution of transcription factor binding properties (for example, differences in the YRE preferred by ScYap1p and Cgap1p); and the versatility and the fast evolution of the structure of the transcription regulation networks (for example, the apparent sharing of Yap1p function between other transcription factors in *C. glabrata*). This model was recently supported by a similar study conducted on the mating/pseudohyphal growth regulation system in yeasts [56] and by an experimental analysis of Mcm1p genomic binding loci over three distant yeast species [57]. All these works concluded the occurrence of a very fast divergence of promoter structure and regulatory network combinatorial circuits, which created a complex equilibrium between the conservation of essential functions and the emergence of new properties. These observations address the role of the evolution of transcriptional networks in the adaptation of yeast species to specific ecological niches. These features could not have been predicted from genome sequences alone and demonstrate the need to combine accurate functional genomic analyses and sequence resources for a larger set of evolutionarily different organisms.

## Materials and methods

### Yeast strains, growth conditions and YRE mutagenesis

The *S. cerevisiae *strain is BY4742 from the Euroscarf collection. The wild-type *C. glabrata *strain used in the kinetic experiments was the sequenced strain CBS418. The *C. glabrata CgAP1Δ *strain and its isogenic wild type were a gift from J Bennett [37]. The *S. cerevisiae *strain expressing Cgap1p in place of ScYap1p was derived from the BY4742 *YAP1*::KanMX strain (Euroscarf). This strain was transformed with a DNA fragment containing the *CgAP1 *ORF fused to the selective marker gene *his5 *from *Schizosaccharomyces pombe*, flanked by about 40 bp corresponding to the regions immediately upstream and downstream of the *YAP1 *ORF, as described previously [58]. The clones having integrated this fragment in place of KanMX were selected on CSM-HIS plates and controlled by PCR and sequencing. The CgAP1-his5 fusion was obtained as follows: CgAP1 was amplified by PCR from *C. glabrata *CBS418 genomic DNA, using oligonucleotides so that a *Sac*II restriction site was introduced 3'. *His5 *was amplified by PCR from a plasmid previously described [58], with oligonucleotides so that a *Sac*II restriction site was introduced 5'. After *Sac*II digestion, the two PCR fragments were ligated using the Quick Ligation kit (New England Biolabs, Ipswich, MA, USA). The cassette for the chromosomic insertion of *CgAP1*-*his5 *was obtained by PCR using oligonucleotides containing sequences flanking the *YAP1 *ORF. The *ScFLR1 *and *CgFLR1 *were amplified from genomic DNA by PCR, using oligonucleotides designed to introduce *Not*1 and *Sac*II restriction sites 3' and 5' of the PCR product, respectively. After *Not*1 and *Sac*II digestions, these PCR fragments were cloned in the plasmid pZLG (Garcia *et al*., in preparation), which contained *lacZ *cloned downstream of the *Sac*II and *Not*1 sites in the polylinker and the *URA3 *selective marker gene. The mutagenesis of YRE and CgYRE in these promoters were conducted using specific oligonucleotides and the QuickChange II Multisite-directed Mutagenesis kit (Stratagene, La Jolla, CA, USA). All constructs were controlled by sequencing. All the oligonucleotides used are described in Additional data file 11. All PCR amplifications were conducted using the Pfx platinium (Invitrogen, Carlsbad, CA, USA) enzyme and the corresponding protocol. Cells were grown in YPD rich media (2% glucose, 1% bactopeptone, 1% yeast extract).

### Transcriptome analyses

#### Time-course experiments and microarray hybridizations

Cells were grown to an OD_600nm _of 0.6 and treated with benomyl (Sigma-Aldrich, St. Louis, MO, USA) to a final concentration of 20 μg/ml (stock solution: 10 mg/ml in DMSO). For mock treatment, cells were incubated with a similar volume of DMSO. The cells were snap-frozen in cold ethanol (final concentration 70% at -80°C) after 2, 4, 10, 20, 40 and 80 minutes of benomyl or mock treatment. RNA was extracted as previously described [26]. Total RNA (10 μg) was used for fluorescent cDNA synthesis according to the amino-allyl protocol. The labeled cDNA was purified and hybridization carried out according to the protocol available from [59]. At least three independent experiments were performed for each time point, using dye switching techniques. The budding yeast arrays were custom-made and contained probes for all yeast ORFs, spotted in duplicate onto Corning Ultragap slides(Corning, NY, USA). The *Candida *arrays were obtained from the Pasteur Institute and contained probes for most of the ORFs from *C. glabrata*, spotted singly onto Corning Ultragap slides at the transcriptome platform [60]. Note that all the microarray data have also been submitted to the Gene Expression Omnibus (GEO) database [61]. The accession number is GSE10244.

#### Image analyses and data processing

The microarrays were read with a Genepix 4000B scanner (Axon. Downingtown, PA, USA) and analyzed with Genepix 6.0 software. Artifactual and saturated signal spots were eliminated. After image quantification, data were normalized over all features with print-tip lowess, using the R/BioConductor packages 'limma' and 'marray' available from [62]. Expression values for replicated spots on the array were averaged. The SAM algorithm [29] in the 'samr' package of R [63] was used to identify genes displaying a change in expression over time, using an equivalent false discovery rate (less than 5%) for all time points. As an additional filter, only genes with smooth expression profiles were retained. These genes displayed a significant change in expression over at least two successive time points. Gene expression patterns with more than two missing values (33%) were also excluded from subsequent analysis. The remaining missing values were replaced by the KNN-imputation [64] method, with the K parameter fixed at 30, as recommended by de Brevern *et al. *[65].

#### Real-time, quantitative RT-PCR analyses

Real-time, quantitative RT-PCR analyses were carried out exactly as described previously [66], using a Light Cycler 480 (Roche, Basel, Switzerland). All the experiments were duplicated, using independent clones to average clone-specific effects. *ACT1 *was used as a reference. The sequences of the oligonucleotides used are available in Additional data file 11.

### Bioinformatic analyses

#### Source of sequence data

Complete genome sequences for *S. cerevisiae *and *C. glabrata *were downloaded from the *Saccharomyces *Genome Database [67,68] and Génolevures [69,70] websites, respectively. Promoter sequences located upstream from the ORF were obtained with RSA tools [71] available from [72]. Upstream regions from -600 bases to -1 base were used for regulatory motif searches, by analysis of the direct strand of DNA.

#### Orthology assignments

Orthology relationships were inferred between *S. cerevisiae *and *C. glabrata *genes using the INPARANOID algorithm [30,73] with the default parameters. This algorithm begins by calculating all pairwise similarity scores between the complete sets of protein sequences from the two genomes, using BLAST [74]. The sequence pairs with the best mutual hits are then detected and serve as central points around which additional orthologs from both species are clustered. Finally, overlapping groups are resolved.

#### MatrixREDUCE algorithm

We used the MatrixREDUCE algorithm [31,42] to detect significant PSAMs in promoter sequences. MatrixREDUCE infers the sequence specificity of a transcription factor directly from genome-wide transcription factor occupancy data by fitting a statistical mechanical model for transcription factor-DNA interaction. The source code is freely available online from [75] and was used for analyses of upstream sequences from positions -600 to -1, searching for 1-7 bp motifs (see the documentation available online for more information).

#### Principal component analysis

PCA is a multivariate statistical method allowing a large number of sample datasets to be described in terms of much smaller numbers of principal components, each of which accounts for significant variability in the data but is not correlated with any other component. A complete interpretation of the biplots, given different transformations of the data expression matrix, can be found elsewhere [76]. The analysis was carried out in the statistical computing and graphics environment R [77].

#### Measurement of similarity between gene expression profiles

Methods for analyzing expression data are often based on the implicit hypothesis that genes with similar functions have similar expression profiles across a set of conditions [78]. For computational analysis, it is necessary to transform the intuitive notion of 'similarity' into quantitative measures. Classically, a distance measured between gene expression profiles is applied [79]. In this study, we used 'Euclidian distance' to assess the relationship between two gene expression profiles. If we denote two sets of measurements *x*_*i *_and *y*_*i*_, where *i *is an index from microarray experiment 1 to *n*, the Euclidean distance between the two profiles *X *and *Y *is given by the following equation:

$$d(X,Y)=\sqrt{\sum_{i=1}^{n}{\left(x_{i}-y_{i}\right)}^{2}}$$

The Euclidean distance takes a value between 0 and + ∞;. A value of 0 means that the two profiles are perfectly superimposed.

#### Hierarchical clustering

The hierarchical clustering procedure has been described in detail elsewhere [79]. It can be summarized by the following five steps: step 1, distances between all genes pairs are calculated, using Euclidean distance, for example (see the previous paragraph); step 2, the resulting distance matrix is thoroughly inspected to find the smallest distance between expression profiles; step 3, the corresponding genes are joined together in the tree and form a new cluster; step 4, the distances between the newly formed cluster and the other genes are recalculated; step 5, steps 2, 3 and 4 are repeated until all genes and clusters are linked in a final tree. We used the 'hclust' function, available in the R programming language, with the 'ward' method for gene agglomeration. Hierarchical clustering results were visualized by representing the ordered expression profiles with a color code [80]: green for negative expression measurements (down-regulated genes) and red for positive expression values (up-regulated genes).

#### Differential clustering algorithm

The DCA was first described by Ihmels *et al*. [10] and the underlying principle is illustrated in Additional data file 3. We applied the DCA to orthologous gene pairs defined by INPARANOID. Genes belonging to one of the two species (the 'reference' yeast) were first classified using the hierarchical clustering method described above. This generated clusters of genes coexpressed in the reference yeast but not necessarily in the other yeast (the 'target' yeast). We then reordered the orthologous counterparts of the genes within each coexpressed cluster in the target yeast using a secondary hierarchical clustering step. DCA results are presented as rearranged distance matrices for each yeast species, with lines and columns ordered according to primary and secondary clustering results. These matrices are of the same dimension (that is, the number of orthologous genes) and are composed of all pairwise distances between gene expression profiles, represented using the following color code: red for small distances (that is, gene pairs strongly coexpressed) and yellow for large distances (that is, gene pairs weakly coexpressed). Finally, the distance matrices were combined into a single matrix, in which each triangle corresponded to one of the distance matrices. This ingenious graphical representation facilitates the intuitive extraction of differences and similarities in the coexpression patterns of the two yeasts, resulting in the definition of four categories of gene clusters: full, partial, split or no conservation of expression. Labels for cluster conservation are based on three correlation measures (C_*a*_, C_*b*_, and C_*ab*_), corresponding to the mean correlations of genes within secondary clusters '*a*' (C_*a*_) and '*b*' (C_*b*_) in the target yeast (see main text) and between these clusters (C_*ab*_). If C_*a*_, C_*b *_and C_*ab *_are higher than a threshold T chosen heuristically (T = 0.3 in this study), the cluster is considered to display full conservation; if (C_*a *_and C_*b*_) > T and C_*ab *_< T, the cluster is considered to display split conservation; if (C_*a *_or C_*b*_) > T, the cluster is considered to display partial conservation; and, if (C_*a *_and C_*b*_) < T, the cluster is considered to display no conservation. The R programming language [77] was used for the DCA approach and graphical representation.

## Abbreviations

BRE: benomyl response element; DCA: differential clustering algorithm; DMSO: dimethylsulfoxide; ORF: open reading frame; PC: principal component; PCA: principal component analysis; PSAM: position apecific affinity matrix; SAM: significance analysis of microarrays; STRE: stress response element; YRE: Yap1 response element.

## Authors' contributions

GL conceived and performed all the bioinformatic analyses and drafted the manuscript, VT performed microarray experiments, CG participated in microarray analyses, CE and CJ contributed to discussions, and FD supervised microarray experiments, performed experimental assays of ScYap1p and Cgap1p activities *in vivo *and drafted the manuscript. All authors read and approved the final manuscript.

## Additional data files

The following additional data are available with the online version of this paper. Additional data file 1 is a document giving the Pearson correlation values between expression measurements obtained for different time points, using orthology relationships between all genes in *S. cerevisiae *and *C. glabrata*. Additional data file 2 is a table listing the genes significantly up- and down-regulated in *S. cerevisiae *and *C. glabrata*, with expression measurements. Additional data file 3 is a document describing the principle of the differential clustering algorithm. Additional data file 4 is a document describing the different transcriptional modules identified with DCA. Additional data file 5 is a table with complete lists of genes in each DCA cluster. Additional data file 6 is document giving the detailed results obtained with the MatrixREDUCE algorithm. Additional data file 7 is table with complete gene lists of up-regulated genes with their associated regulatory controls (ScYap1p-dependant genes, Cgap1p-dependant genes or other regulatory controls). Additional data file 8 is a document giving an overview of the methods used in this study. Additional data file 9 is a document describing a comparative genomic analysis of YREs in yeasts. Additional data file 10 is a document describing the case of the *FLR1 *gene, in which the conservation of oxidative stress response deals with the divergence of *cis*-regulatory sequences and of regulatory network structure. Additional data file 11 is a table listing oligonucleotides used to construct the mutated yeast strains shown in Figure 4. Additional data file 12 is a document describing the mutagenesis of the YRE and CgYRE motifs.

## Supplementary Material

Additional data file 1 Pearson correlation values between expression measurements obtained for different time points, using orthology relationships between all genes in *S. cerevisiae *and *C. glabrata*. Click here for file

Additional data file 2 Genes significantly up- and down-regulated in *S. cerevisiae *and *C. glabrata*, with expression measurements. Click here for file

Additional data file 3 Principle of the differential clustering algorithm. Click here for file

Additional data file 4 Different transcriptional modules identified with DCA. Click here for file

Additional data file 5 Genes in each DCA cluster. Click here for file

Additional data file 6 Results obtained with the MatrixREDUCE algorithm. Click here for file

Additional data file 7 Up-regulated genes with their associated regulatory controls (ScYap1p-dependant genes, Cgap1p-dependant genes or other regulatory controls). Click here for file

Additional data file 8 Overview of the methods used in this study. Click here for file

Additional data file 9 Comparative genomic analysis of YREs in yeasts. Click here for file

Additional data file 10 Description of the *FLR1 *gene, in which the conservation of oxidative stress response deals with the divergence of *cis*-regulatory sequences and of regulatory network structure. Click here for file

Additional data file 11 Oligonucleotides used to construct the mutated yeast strains shown in Figure 4. Click here for file

Additional data file 12 Mutagenesis of the YRE and CgYRE motifs. Click here for file
