# Supplementary material for: Genome adaptation to chemical stress: clues from comparative transcriptomics in Saccharomyces cerevisiae and Candida glabrata
Source: Genome Biol. 2008 Nov 24;9(11):R164. doi: 10.1186/gb-2008-9-11-r164 (PMC2614496; doi:10.1186/gb-2008-9-11-r164)
Supplement: Additional data file 1 — Pearson correlation values between expression measurements obtained for different time points, using orthology relationships between all genes in S. cerevisiae and C. glabrata. [file gb-2008-9-11-r164-S1.pdf]

<u>Additional data File 1</u>: Cross-species correlation values between different time points.

Using orthology relationships between all genes in *S. cerevisiae* and *C. glabrata*, we calculated the Pearson correlation values between expression measurements obtained in different time points. Results are presented in Figure S1A (below). We could observe that each *C. glabrata* time point exhibited its maximal correlation measure with a later *S. cerevisiae* time point. For instance the time point 2 minutes in *C. glabrata* displayed its maximal correlation value with the time point 10 minutes in *S. cerevisiae*; the time point 4 minutes in *C. glabrata* displayed its maximal correlation with the time point 40 minutes in *S. cerevisiae*; and so on… (See the Figure S1B for a graphical representation of the maximal correlation measures between *C. glabrata* and *S. cerevisiae* time points). Such an observation agree with PCA results (see main text and Figure 1) and show that the transcriptional response to benomyl stress was faster in *C. glabrata* than in *S. cerevisiae*.

A		*C. glabrata*					
		Time 1 (2min)	Time 2 (4min)	Time 3 (10min)	Time 4 (20min)	Time 5 (40min)	Time 6 (80min)
S. cerevisiae	Time 1 (2min)	0.05	0.08	0.12	0.09	0.08	0.07
	Time 2 (4min)	0.18	0.19	0.16	0.11	0.08	0.14
	Time 3 (10min)	0.21	0.26	0.27	0.23	0.18	0.2
	Time 4 (20min)	0.2	0.27	0.39	0.36	0.3	0.23
	Time 5 (40min)	0.19	0.3	0.52	0.57	0.52	0.27
	Time 6 (80min)	0.13	0.26	0.47	0.58	0.63	0.36

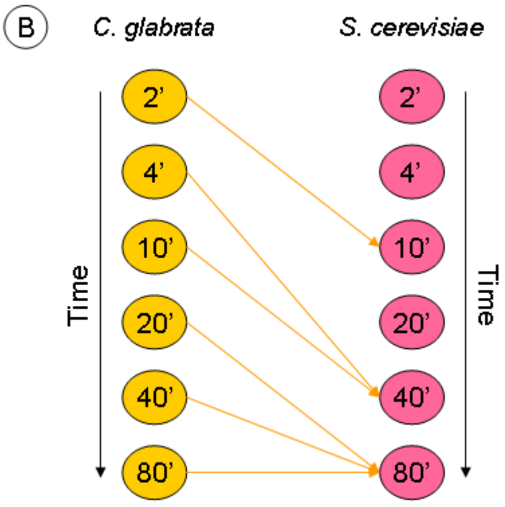

<u>Figure legend S1</u>: (A) Correlation values between expression data in the two different species (after orthology assignement). Maximal correlation values between *C. glabrata* time points and time points in *S. cerevisiae* are coloured in yellow. (B) Graphical representation of the relationships between the time points in the two species studied here. In each species, the time point expression measurements are represented by nodes and arrows connect experiments with the highest correlation values.
