# Supplementary material for: Genome adaptation to chemical stress: clues from comparative transcriptomics in Saccharomyces cerevisiae and Candida glabrata
Source: Genome Biol. 2008 Nov 24;9(11):R164. doi: 10.1186/gb-2008-9-11-r164 (PMC2614496; doi:10.1186/gb-2008-9-11-r164)
Supplement: Additional data file 3 — Principle of the differential clustering algorithm. [file gb-2008-9-11-r164-S3.pdf]

Additional data file S3: Principle of the differential clustering algorithm (DCA).

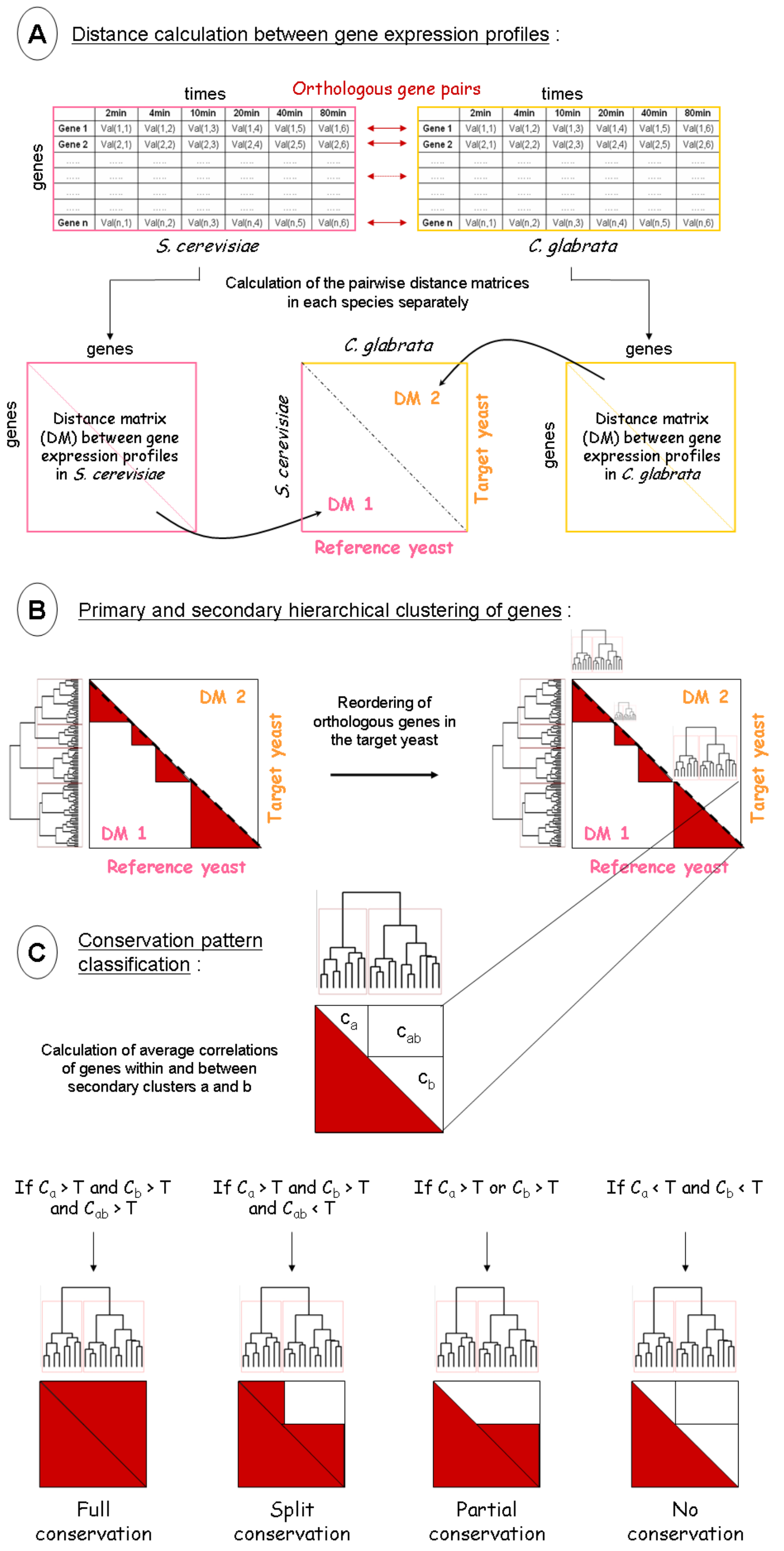

Figure legend S3: A complete description of the DCA can be found in the article by Ihmels *et al*. (Ihmels et al., 2005a). (A) The DCA is applied to a set of orthologous genes for which

expression has been monitored in each species. Gene expression profiles are first ordered according to the orthologous relationships between genes. Complete distance matrices (DMs) are calculated independently in each species, using Euclidean distances (see Method in the main text). These distance matrices are represented with the following color code: red for small distances and white for large distances. As the obtained DMs are of the same dimension (the number of orthologous genes), they are combined into a single matrix, in which each triangle corresponds to one of the initial DMs. At this stage, a reference and a target species are identified. (B) Genes belonging to the reference species are classified by primary hierarchical clustering. Clusters of genes coexpressed in the reference yeast are thus obtained and, within each coexpressed subgroup, genes in the target yeast are classified by secondary hierarchical clustering. At each stage of primary and secondary clustering, orthologous genes are arranged in the same order in both yeasts. (C) The final clusters are classified into different conservation classes corresponding to "*full*", "*partial*", "*split*" and "*no*" conservation. For this, each cluster is subdivided into two secondary clusters, referred as "*a*" and "*b*". The cluster is then characterized in terms of three correlation values, corresponding to the mean correlations of genes within (C_a and C_b) and between (C_{ab}) clusters "a" and "b". These correlation values are then compared with a specific threshold value, T, to determine the conservation class to which the cluster is assigned.
