# Supplementary material for: Genome adaptation to chemical stress: clues from comparative transcriptomics in Saccharomyces cerevisiae and Candida glabrata
Source: Genome Biol. 2008 Nov 24;9(11):R164. doi: 10.1186/gb-2008-9-11-r164 (PMC2614496; doi:10.1186/gb-2008-9-11-r164)
Supplement: Additional data file 4 — Different transcriptional modules identified with DCA. [file gb-2008-9-11-r164-S4.pdf]

<u>Additional data file 4</u>: Description of the different transcriptional modules identified with DCA.

Note that functional category analyses were based on gene ontology (GO) information and p-values were calculated using GOTermFinder (Boyle *et al.,* 2004). Complete lists of genes in each cluster are available in Additiona data file 5.

Description of downregulated conservation clusters. Clusters 1, 2, and 3 comprised 387 orthologous gene pairs displaying clearly downregulated expression in *S. cerevisiae* (Figure 2A, green segments). As expected from previous analyses of stress responses in *S. cerevisiae*, most of these genes are involved in regulating various aspects of cytosolic translation, such as tRNA processing (p=7.15 x 10-8), rRNA processing (p=1.37 x 10-45), translation initiation (p=1.29 x 10-5) and RNA polymerase I transcription (p=6.17 x 10-05), or encode proteins involved in ergosterol biosynthesis (p=9.17 x 10-8) (Figure S4). These three clusters had very similar expression profiles in *S. cerevisiae*. Thus, clusters 1, 2 and 3 may not be independent transcriptional modules, and may instead account for subtle differences in the regulation of a huge cluster of corepressed genes. Indeed, gene ontology (GO) categories were equally distributed among the three clusters, except for ergosterol pathway genes, which mostly belonged to cluster 3, and genes encoding RNA polymerase I subunits and rRNA modification factors, most of which were found in cluster 1. Interestingly, clusters 1, 2 and 3 were differently annotated by DCA (clusters 1 and 3 are "*partially conserved*" and cluster 2 is "*fully conserved*"), indicating that different situations apply to these three clusters. In clusters 1 and 3, most of the genes in the non-conserved parts (1a and 3a) were found to have completely lost their corepression properties in *C. glabrata,* whereas the genes in part 2b of cluster 2 displayed expression patterns similar to those of the genes in cluster 2a. Further experiments are required to determine whether this apparent lower sensitivity to drug repression corresponds to a divergence of the function of these genes in *C. glabrata*.

Description of split conservation clusters. Split conservation clusters 4 and 5 comprised 143 genes with invariant expression in *S. cerevisiae*. As a consequence, GO searches gave results of low significance for these clusters. Nevertheless, these orthologous gene pairs were selected for DCA analyses because they presented modified expression profiles in *C. glabrata*. This accounts for these two clusters being annotated as "*split conservation*" clusters, as they included genes that were either up- or downregulated in *C. glabrata*. The upregulated

C. glabrata genes were orthologs of *S. cerevisiae* genes involved in oxidative stress responses, including *TRX3*, *TRX1*, *TSA1*, *ZWF1* and *YAP1*, which encodes the major transcriptional regulator of the oxidative stress response. This list also included the RPN4 gene, which encodes the major transcriptional regulator of proteasome activity. As all these genes have previously been shown to be coinduced by oxidative stress due to metals and metalloids in *S. cerevisiae* (Haugen *et a.*, 2004), the difference observed here may not be due to a major difference in the gene expression networks of the two organisms. Instead, it probably reflects a difference in the sensing and sensitivity of these pathways to the dose of benomyl used in these experiments.

Description of upregulated conservation clusters. Clusters 6, 7 and 8 were upregulated (Figure 2A, red segments). Together, they comprised 188 orthologous gene pairs displaying an upregulation of expression in response to benomyl in *S. cerevisiae*. Most of these genes are involved in sulfur metabolism (p=1.63 x 10-11), the response to chemical stress (p=4.13 x 10-9) or carbohydrate metabolism (p=5.26 x 10-5) (Figure S4). By contrast to observations for the downregulated clusters, clusters 6, 7 and 8 had different gene expression profiles. Cluster 7 genes were induced very early, cluster 8 genes were induced at an intermediate time point and cluster 6 genes were induced late. The coinduction of the genes in cluster 7 was remarkably conserved in *C. glabrata*. This cluster contained genes involved in stress responses, such as *FLR1*, *ECM4*, *YML131W* and *GRE2*, or methionine metabolism, such as *MET2* and *MET16*. Based on YEASTRACT information (Teixeira *et al.*, 2006), 92% of these genes are known targets of the transcription factor Yap1p in *S. cerevisiae* and 67% are regulated by the transcription factors Msn2p/Msn4p. Moreover, the most highly conserved part of cluster 8 (cluster 8a) also contained mostly Yap1p targets (94%). Most of the genes in the conserved part of cluster 6 (6b) were found to be involved in sulfur amino acid metabolism or carbohydrate metabolism. This part of cluster 6 contained target genes of Yap1p (54%), Met4p (42%) and Msn2p (38%). We found no significant ontology term for genes in the non conserved part of cluster 6 (6a). Thus, the most conserved parts of the transcriptional networks induced by benomyl in *S. cerevisiae* and *C. glabrata* contained a high proportion of Yap1p target genes (e.g. *FLR1*, *GRE2*, *OYE2*, *ERO1*). Furthermore, the MET genes and many of the Msn2p/Msn4p target genes involved in chaperoning (HSP genes) or carbohydrate metabolism also belonged to conserved clusters.

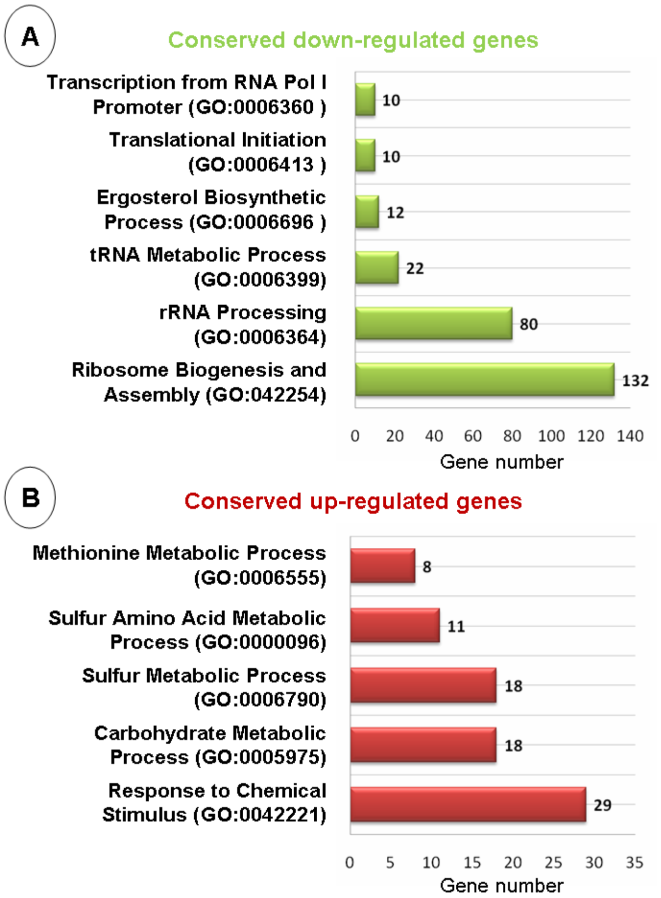

Figure legend S4: Functional characterization of genes with conserved patterns of coexpression between *S. cerevisiae* and *C. glabrata*. Genes belonging to coexpressed clusters (or subclusters) identified with the DCA (Figure 2A) as conserved in both yeasts were analyzed using the Gene Ontology data available for *S. cerevisiae*. (A) Significant sharing of GO terms identified in conserved downregulated genes (DCA cluster 2 and subclusters 1b and 3b). (B) Significant sharing of GO terms identified in conserved upregulated genes (clusters 7 and 8 and subcluster 6b). Downregulated clusters comprised mostly genes involved in various aspects of cytosolic translation regulation, whereas upregulated clusters comprised mostly genes involved in responses to chemical stress and metabolic processes (see the previous text for a more detailed description of the functional categories presented here).
