# Supplementary material for: Genome adaptation to chemical stress: clues from comparative transcriptomics in Saccharomyces cerevisiae and Candida glabrata
Source: Genome Biol. 2008 Nov 24;9(11):R164. doi: 10.1186/gb-2008-9-11-r164 (PMC2614496; doi:10.1186/gb-2008-9-11-r164)
Supplement: Additional data file 6 — Results obtained with the MatrixREDUCE algorithm. [file gb-2008-9-11-r164-S6.pdf]

<u>Additional data file 6</u>: PSAMs identified with MatrixREDUCE.

To detect significant PSAM (position-specific affinity matrices) in promoter sequences of genes whose expression was measured during benomyl stress response, we used the MatrixREDUCE algorithm. This algorithm takes microarray data and promoter sequences as input. Expression data for *S. cerevisiae* and *C. glabrata* were analysed independently, using for both yeasts, upstream sequences from positions from -600 to -1 and searching for 1-7bp motifs. The identified PSAMs were transformed into affinity logo for visualization, the ten most significant are shown Figure S6.1 (*S. cerevisiae* results) and Figure S6.2 (*C.glabrata* results).

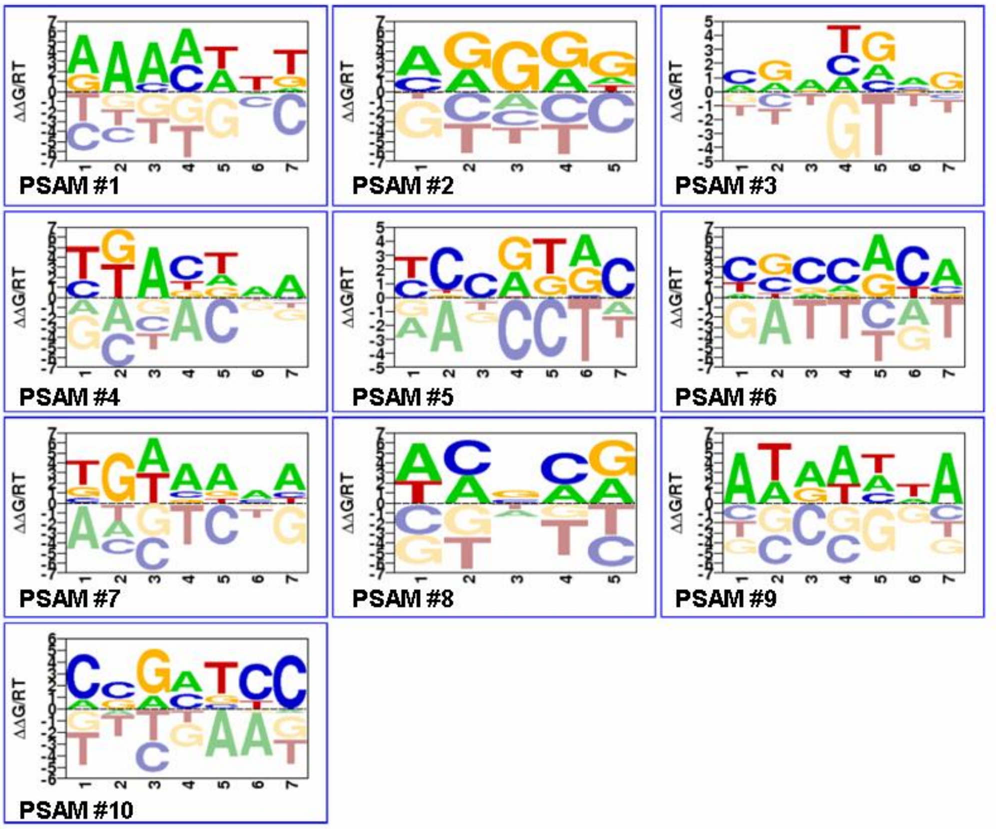

<u>Figure legend S6.1</u>: Images of the PSAMs identified in *S. cerevisiae* using MatrixREDUCE.

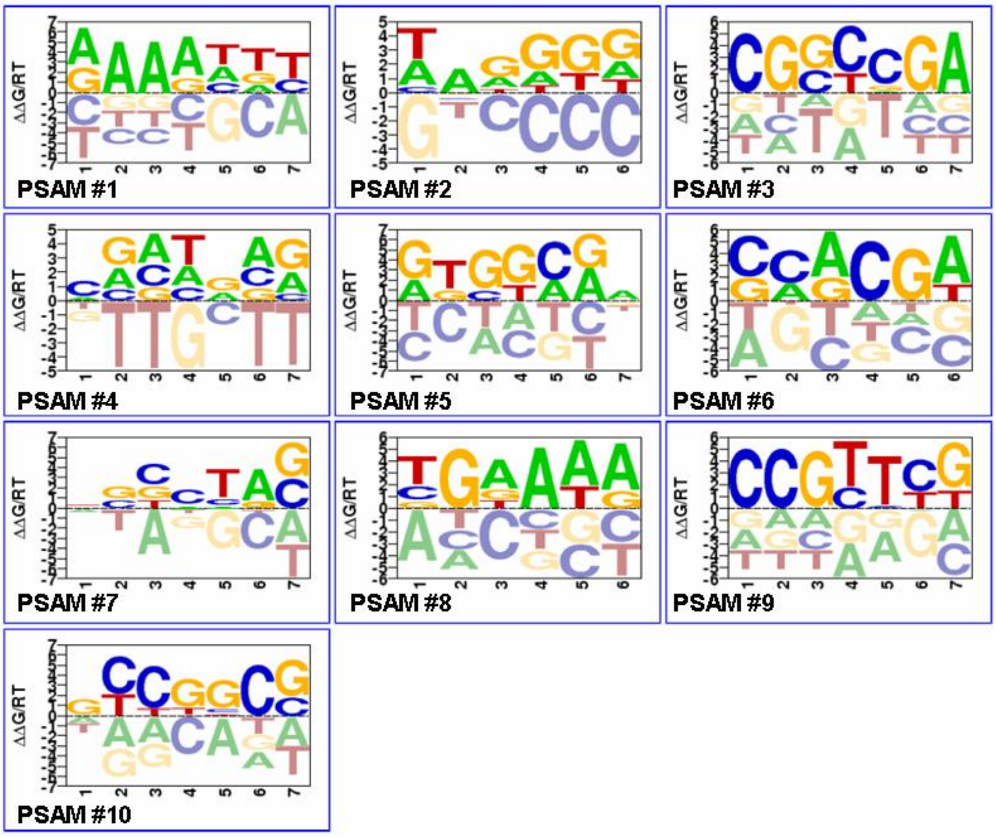

Figure legend S6.2: Images of the PSAMs identified in *C. glabrata* using MatrixREDUCE.

To compare PSAMs obtained respectively in *S. cerevisiae* and *C. glabrata*, we calculated Pearson correlation values between the $\Delta\Delta G$ values for each nucleotide at each position, for every possible overlap of every PSAM pairs. Maximal correlation values are presented in Table S6.3 (below). Correlation values coloured in yellow correspond to the three pairs of PSAMs presented in Figure 2B. As pointed in the main text, none of the PSAM identified in *C. glabrata* exhibited significant correlation (higher than 0.6) with the *S. cerevisiae* PSAM #4, whose consensus sequence T(G/T)ACTAA is compatible with the Yap1p Response Element (YRE). These values are lower than 0.3 and are underlined in grey (Table S6.3).

		C. glabrata									
		PSAM #1	PSAM #2	PSAM #3	PSAM #4	PSAM #5	PSAM #6	PSAM #7	PSAM #8	PSAM #9	PSAM #10
S. cerevisiae	PSAM #1	0.69	0.26	-0.16	0.12	-0.05	0.12	-0.04	-0.15	-0.13	-0.43
	PSAM #2	0.29	0.71	-0.06	0.19	0.26	0.49	-0.27	0.26	-0.5	-0.26
	PSAM #3	-0.22	-0.14	0.49	0.6	-0.05	0.2	-0.07	0.08	0	-0.22
	PSAM #4	-0.31	0.19	0.18	0.07	-0.18	-0.24	-0.29	0.27	-0.05	-0.27
	PSAM #5	-0.18	0.38	-0.17	0.23	-0.06	0.15	0.17	0.35	-0.07	0.44
	PSAM #6	-0.46	-0.16	0.29	0.27	-0.29	0.08	-0.16	0.28	0	0.13
	PSAM #7	-0.02	-0.13	-0.02	-0.01	-0.31	0.2	-0.23	0.74	-0.42	-0.35
	PSAM #8	0.41	0.28	0.23	0.39	0.51	0.25	-0.14	-0.17	-0.58	-0.18
	PSAM #9	0.34	0.19	-0.16	-0.01	0.15	-0.16	-0.21	0.41	-0.08	-0.35
	PSAM #10	-0.08	-0.42	0.21	0.28	-0.33	0.23	0	-0.23	0.38	0.05

Table S6.3: Pearson correlation values calculated between all pairs of PSAMs identified in *S. cerevisiae* and *C. glabrata*. Correlation values corresponding to the three pairs of PSAMs shown Figure 2B are coloured in yellow. The grey line indicates that no significant correlations were observed between the *S. cerevisiae* PSAM #4 and the *C. glabrata* PSAMs.
