# Supplementary material for: Genome adaptation to chemical stress: clues from comparative transcriptomics in Saccharomyces cerevisiae and Candida glabrata
Source: Genome Biol. 2008 Nov 24;9(11):R164. doi: 10.1186/gb-2008-9-11-r164 (PMC2614496; doi:10.1186/gb-2008-9-11-r164)
Supplement: Additional data file 8 — Overview of the methods used in this study. [file gb-2008-9-11-r164-S8.pdf]

Additional data file 8: Overview of the methods used in this study.

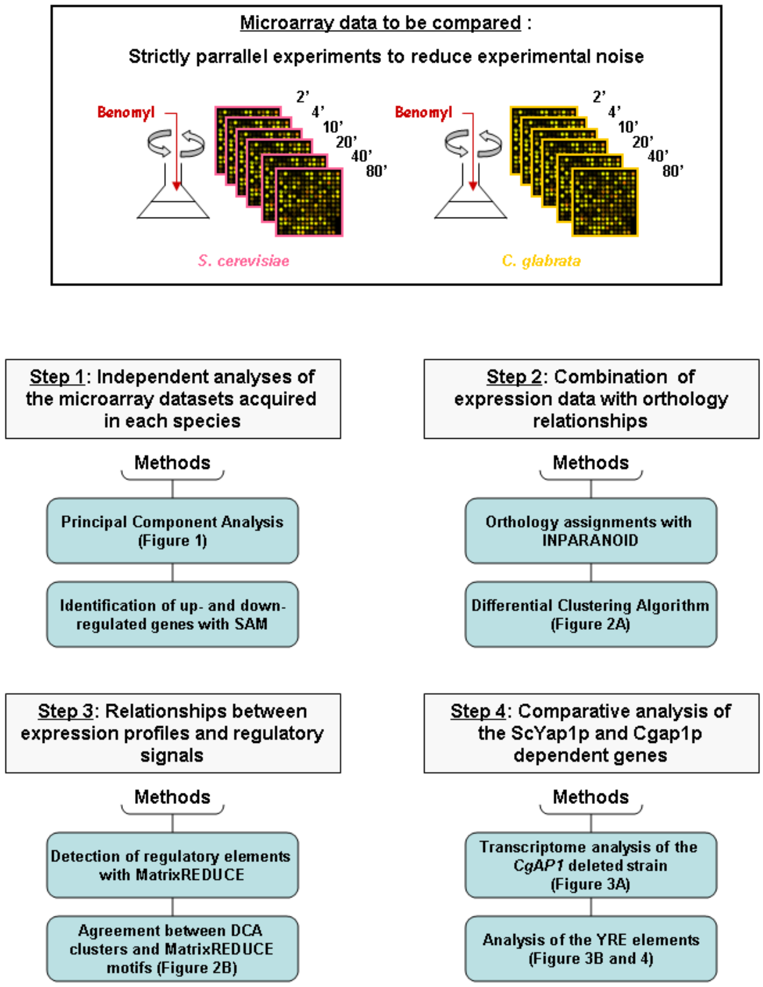

Figure legend S8: We proposed a general protocol for studies of the evolution of genetic networks involved in similar biological processes. We optimized conditions for the integration of expression data into a cross-species comparison by (*i*) chosing species from the same phylum and with a high rate of orthologous genes; (*ii*) producing experimental data as comparable as possible between species; (*iii*) sequentially applying a set of complementary bioinformatic approaches to assess the validity of the results.
