# Supplementary material for: Genome adaptation to chemical stress: clues from comparative transcriptomics in Saccharomyces cerevisiae and Candida glabrata
Source: Genome Biol. 2008 Nov 24;9(11):R164. doi: 10.1186/gb-2008-9-11-r164 (PMC2614496; doi:10.1186/gb-2008-9-11-r164)
Supplement: Additional data file 9 — Comparative genomic analysis of YREs in yeasts. [file gb-2008-9-11-r164-S9.pdf]

<u>Additional data file 9</u>: Comparative genomic analysis of Yap1 Response Element (YRE) in yeasts.

We chose three *Saccharomyces sensu stricto* yeast species (*S. paradoxus*, *S. mikatae* and *S. bayanus*) and a more evolutionary distant yeast species, *Candida albicans* (Figure S9, A). Orthology relationships between *Saccharomyces* species were obtained from the work of Kellis *et al.* (2003), and orthology relationships between *Candida albicans* and *S. cerevisiae* were download from the Candida Genome Database.

Taking *S. cerevisiae* as reference yeast, we analyzed the loss of YREs in promoter of orthologous genes. We considered 207 genes in *S. cerevisiae* that were (*i*) targets of ScYap1p and (*ii*) contained at least one YRE in their promoter. From these 207 genes, 101 had an orthologous counterpart in *C. glabrata* in which the YREs were completely lost. Analysing promoter sequences of the corresponding orthologous genes belonging to the *Saccharomyces sensu stricto* evolutionary tree, we could observed a progressive decrease in the number of promoters that contained at least one YRE: from 100% in *S. cerevisiae* to 66% in *S. paradoxus*, 63% in *S. mikatae* and finally 50% in *S. bayanus*. Sixty percent of these promoters contained an YRE in *C. albicans*.

We analyzed in more details the changes in YRE frequency between *S. cerevisiae* and *C. glabrata*. From the 207 genes in *S. cerevisiae* that were (*i*) targets of ScYap1p and (*ii*) contained at least one YRE in their promoter, 106 retained a Yap1 Response Element (YRE) in the promoter of their orthologs in *C. galabrata*. As the YRE found in promoters of *C. glabrata* genes were not necessarily the same than the YRE initially identified in the promoters of *S. cerevisiae* genes, we calculated the percentage of genes with each type of YRE. Difference between the percentages obtained in *S. cerevisiae* genes and those obtained in their orthologs in other species are presented Figure S9B. We observed that most of the YRE motifs were lost among evolution (the percentage is lower in other yeast species than it is in *S. cerevisiae*), except for the TTACAAA and TGACAAA motifs whose frequencies were clearly higher in *Candida* species (*C. glabrata* and *C. albicans*) than in *S. cerevisiae*. In agreement with results presented Figure 3, the TTACAAA motif appeared to be enriched in promoter of *C. glabrata* genes, compared to those of *S. cerevisiae.* Further analyses are required to precisely reconstruct the evolutionary path leading to the TTACAAA and TGACAAA motif enrichments in *Candida* species.

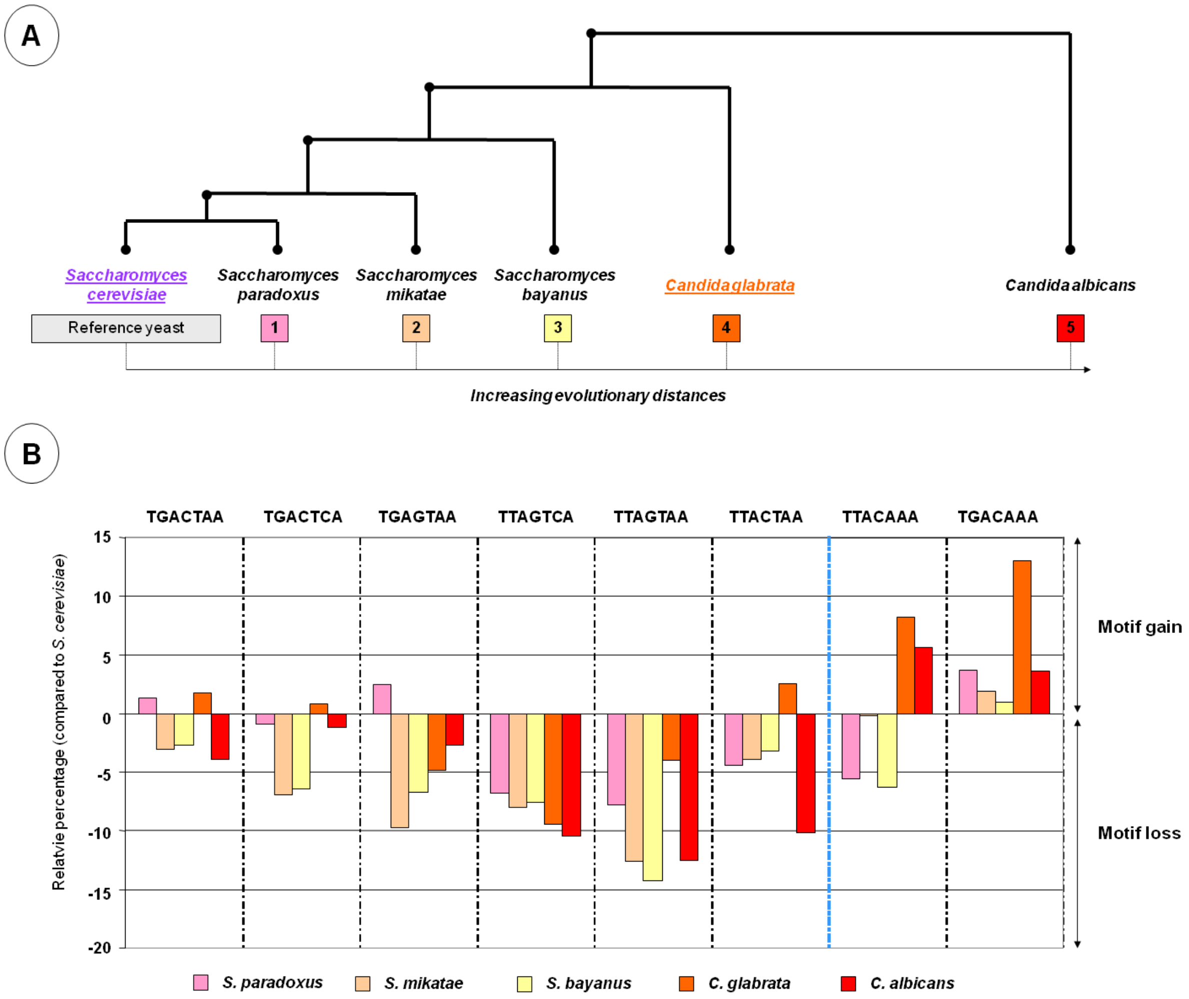

<u>Figure legend S9</u>: Comparative genomic analysis of Yap1 Response Element (YRE) in yeasts**.** (A) Phylogenetic tree of the yeast species used in this study. (B) Relative YRE percentages in promoter of genes that (*i*) are targets of ScYap1p and (*ii*) contained at least one YRE in *S. cerevisae* and *C. glabrata*. Negative values correspond to a relative "motif loss" since the YRE percentage is higher in *S. cerevisiae* than it is in the orthologous genes in other yeast species. Positive values correspond to a "motif gain", the YRE percentage in higher in other yeast species than it is in *S. cerevisiae.*
