# Supplementary material for: Genome adaptation to chemical stress: clues from comparative transcriptomics in Saccharomyces cerevisiae and Candida glabrata
Source: Genome Biol. 2008 Nov 24;9(11):R164. doi: 10.1186/gb-2008-9-11-r164 (PMC2614496; doi:10.1186/gb-2008-9-11-r164)
Supplement: Additional data file 10 — Description of the FLR1 gene, in which the conservation of oxidative stress response deals with the divergence of cis-regulatory sequences and of regulatory network structure. [file gb-2008-9-11-r164-S10.pdf]

<u>Additional data file 10</u>: The conservation of oxidative stress response deals with the divergence of cis-regulatory sequences and of regulatory networks structure: the case of *FLR1*.

The complexity of the evolution of the promoters responding to oxidative stress was nicely exemplified by the *ScFLR1* gene. The cis-regulatory motifs responding to different sources of stress, have been experimentally characterized in this promoter, and in the promoters of its orthologs in *C. glabrata* (*CgFLR1*) and *C. albicans* (*CaMDR1*) (Figure S10). *ScFLR1* contains three YREs (Nguyen *et al.*, 2001). YRE1 is involved only in the methylsulfonate response, whereas YRE2 and YRE3 mostly account for the H_2O_2 (HRE) and benomyl (BRE) responses, respectively. In the promoter sequence of *CgFLR1*, the TTACAAA motif was found in position of the YRE2 and YRE3 position has been converted into its reverse complement motif (TTAGTAA). No equivalent of YRE1 could be identified (Figure S10, below). Our computational and experimental analyses indicated that TTACAAA is a major BRE in *C. glabrata* and that, at least in the case of *CgFLR1*, TTAGTAA has no role in this response (Figure 4A). Interestingly, the TTAGTAA motif is conserved in the promoter of *CaMDR1* (Figure S10), where it acts as a HRE regulated by Cap1p (Rognon *et al.*, 2006), the functional homologue of ScYap1p and Cgap1p, which suggests that it could play a similar role in *C. glabrata*. In contrast, the BRE identified for *CaMDR1* contains no canonical YRE (Figure S10) and is apparently not regulated by Cap1p (Rognon *et al.*, 2006; Riggle *et al.*, 2006). Then, we have here an example of a progressive evolution from a different sharing of the tasks (HRE, BRE) between different cis-elements (TTAGTAA, TTACAAA) regulated by the same transcription factor in *S. cerevisiae* and *C. glabrata*, to the *C. albicans* situation in which the BRE activity has been transferred to a different regulatory pathway. Still, the benomyl response properties of *ScFLR1*, *CgFLR1* and *CaMDR1* are perfectly conserved.

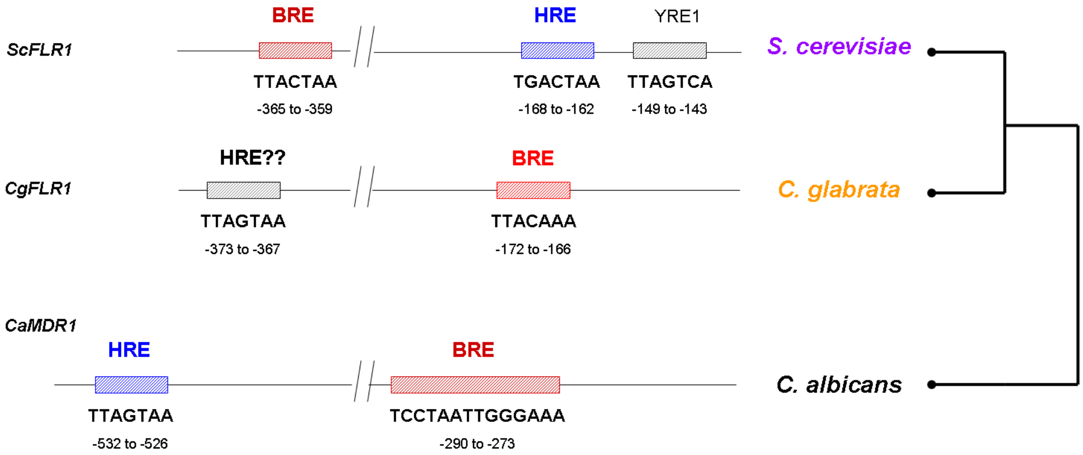

Figure legend S10: Upstream regions of the orthologous genes *ScFLR1* in *Saccharomyces cerevisiae*, *CgFLR1* in *Candida glabrata* and *CaMDR1* in *Candida albicans*. In *S. cerevisiae*, the motifs TTAGTCA, TGACTAA and TTACTAA correspond to YRE1, YRE2 and YRE3, respectively (Nguyen *et al.*, 2001). YRE2 is mostly associated with the H2O2 stress response (HRE), whereas YRE3 is mostly associated with benomyl responses (BRE) (Nguyen *et al.*, 2001). The HRE and BRE in *C. glabrata* and *C. albicans* are indicated, together with the distance of the cis-regulatory sequences from the start codon.
