# Supplementary material for: Genome adaptation to chemical stress: clues from comparative transcriptomics in Saccharomyces cerevisiae and Candida glabrata
Source: Genome Biol. 2008 Nov 24;9(11):R164. doi: 10.1186/gb-2008-9-11-r164 (PMC2614496; doi:10.1186/gb-2008-9-11-r164)
Supplement: Additional data file 12 — Mutagenesis of the YRE and CgYRE motifs. [file gb-2008-9-11-r164-S12.pdf]

Additional data file 12: Mutagenesis of the YRE and CgYRE motifs.

Mutations of YRE and CgYRE motifs in promoter of Sc*FLR1* and Cg*FLR1* genes were obtained using the procedure described in Methods (see the main text). Sequences of motifs before and after the mutagenesis procedure are shown below (Figure S12).

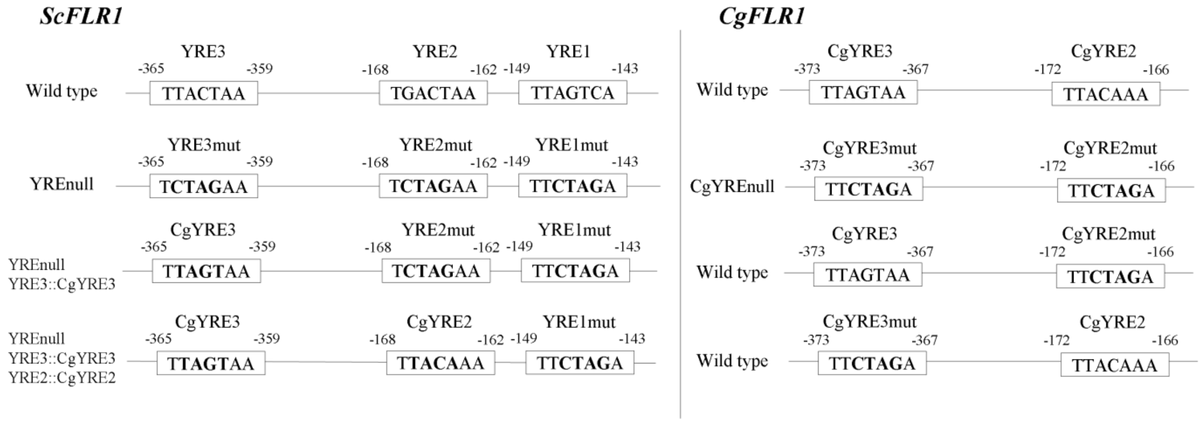

Figure legend S12: Mutagenesis of the YRE and CgYRE motifs.
